# The role of ethnic tourism in the food knowledge tradition of Tyrolean migrants in Treze Tílias, SC, Brazil

**DOI:** 10.1186/s13002-018-0224-9

**Published:** 2018-04-06

**Authors:** Elisabeth Kuhn, Ruth Haselmair, Heidemarie Pirker, Christian R. Vogl

**Affiliations:** Working Group: Knowledge Systems and Innovations, Division of Organic Farming, Department for Sustainable Agricultural Systems, University of Natural Resources and Life Sciences, Vienna, Gregor Mendel Straße 33, 1180 Vienna, Austria

**Keywords:** Culture, Knowledge tradition, Ethnic tourism, Cuisine, Migration

## Abstract

**Background:**

Food knowledge and consumption in the context of migration is an important topic in ethnobiological research. Little research is done on the process of how external factors impact food knowledge amongst migrants. Taking into account social organisation and power relations of food knowledge transmission and distribution of food knowledge, this study sheds light on how the accessibility of resources, the predominant cuisine in the host country and ethnic tourism influences the food knowledge tradition of Tyrolean migrants and their descendants in Treze Tílias.

**Methods:**

Field research was conducted in Austria and Brazil in 2008–2009, using free-listing, social network analysis and participatory observation. The collected data was analysed by calculating Smith’s Salience index, visualising personal and social networks and qualitative text analysis.

**Results:**

Tyroleans in Austria had a different perception and a higher agreement of what Tyrolean food comprises than Tyroleans in Brazil, indicating different developments: Tyrolean migrants adapted their food habits according to available resources and over time in Brazil. Later, ethnic tourism had a strong impact: In Treze Tílias, dishes with the highest Smith’s Salience index—forming the core of cultural food knowledge—strongly coincided with Tyrolean food served in ethnic restaurants, whose staff were perceived to be experts in Tyrolean food.

**Conclusion:**

Despite most food knowledge in Treze Tílias was transmitted within families, ethnic food prepared in restaurants and hotels determined the shared perception of what Tyrolean food comprises. Perceived as experts, the staff in ethnic restaurants were in a powerful position to transform cultural food knowledge by providing institutionalised and standardised knowledge about Tyrolean food.

## Background

### Migration, food traditions and ethnic tourism

To understand how food knowledge in migrant societies is maintained and transformed, it is necessary to go back to where food knowledge is learned: children learn about food when they first come into contact with the flavours and textures of the food eaten by the members of the group to which they belong. “Although taste remains something distinctive and individual, it is imprinted from birth with the stamp of a culture” [1]. Children learn the norms and conventions of their society about what is edible, which foods are taboo and when a certain type of food should be eaten. These eating habits are deeply rooted in the value system of the individual and people find it difficult to fundamentally transform their eating habits and only do so very slowly [2]. Therefore, migrants attempt to preserve their eating habits and maintain their food traditions for a considerable time [3].

Derived from the Latin word ‘tradere’, tradition refers to everything that is transmitted from the past to the present [4]. Through traditions, people attempt ‘to establish continuity with a suitable historic past’ [5]. Traditions are not static, but are constantly imbued ‘with dynamic content and interpretation’, being a ‘conscious model of past lifeways that people use in the construction of their identity’^1^ [6]. Through traditions—including food traditions—migrants connect with their collective past and establish a link with their ancestors’ homeland [3]. Thus, the term ‘traditional food’ describes an emic perspective of the migrants on their food [7]. The consumption of traditional food strengthens the social coherence of the migration group, and at the same time distinguishes the migrants from the rest of society in the host country [8].

The maintenance of the migrants’ own traditions not only has an effect on individual migrants and the migrant community, but on the host country as well. Yang and Wall [9] note that some host countries ‘take advantage of their cultural diversity and employ ethnic tourism to stimulate local economic development’. This is happening in the Brazilian state of Santa Catarina, for example, where European immigrants, including Germans, Italians and Austrians, have settled. In ethnic tourism, culture is commoditised, while the ‘most marketable forms of cultural exoticism are the more spectacular aspects of the lifestyles and artefacts of minority groups’ [9] such as dance, music, architecture, art and food. In Treze Tílias, the Austrian settlement in Santa Catarina, the local development plan stipulates the strengthening of certain cultural aspects for the purposes of tourism, making it an active part of local politics [10].

When tourists visit locations in which ethnic tourism has been established on the basis of the migrants’ traditions and culture, they can have ‘exotic’ and unfamiliar experiences within their own country. Experiences during visits to such locations can differ considerably from those gained during visits to the migrants’ country of origin, including the culinary experience they have. The range of ethnic food^2^ served in ethnic restaurants is usually limited to a selection of ‘iconic dishes’ in a national culinary repertoire, which is not always representative of the wide range of food in local eating places in the migrants’ country of origin [11]. Bak [12] expresses this process as ‘a form of standardization (homogenization)’ that ‘occurs when a cuisine is introduced to other cultures as ethnic food’. [12].

Even though food is an important tourist attraction [13, 14], Cohen and Avieli [11] remark that ethnic food can become an impediment for tourists due to the different tastes and ingredients, hygiene levels or food taboos. Therefore, food in tourist locations often ‘becomes acceptable only if it is to some extent transformed’ [11]. To be commercially viable, restaurant owners need to be aware of their potential customers’ tastes and their prior exposure to different foodway systems. ‘The anticipated clientele, then, is a major factor in the negotiation of edibility and exoticness, and ethnic restaurants must frequently emphasize the edibility of the exotic in order to attract non-native customers’ [15]. According to Long [15], menus in ethnic restaurants are negotiated by restaurant owners using five basic strategies: framing the culinary experience, naming or translating dishes, explicating, selecting the menu and adapting recipes.

Most studies on food and tourism focus on the tourist experience. Among the few studies that focus on the influence of tourism on the host country cuisine are studies in Taiwan [16], France [17], England [18], Vietnam [19, 20] and Portugal [21]. Specific ethnic food and food practices can be given a higher status due to tourism [16–18, 20]. While food that is significantly altered to suit the taste of tourists can be rejected by the host community, the introduction of ‘authentic’ food in the menus of tourism-oriented restaurants can result in these dishes being given a higher status [19]. Tourism ‘can be an agent of both preservation and disfigurement’ of foodstuffs and foodways, as stated by Texeira [21]. For example in Vietnam, tourism has triggered structural societal changes in some areas, affecting the food knowledge tradition to the extent that cultural food knowledge is lost [20].

This study focused on the impact of ethnic tourism on the host food knowledge tradition. The term ‘knowledge’ as it is used here refers to Barth’s definition of knowledge as ‘what a person employs to interpret and act on the world’. Within the term he includes ‘feelings (attitudes) as well as information, embodied skills as well as verbal taxonomies and concepts: all the ways of understanding that we use to make up our experienced, grasped reality’ [22].

To emphasise the focus on cultural consensus, and therefore on the shared aspects of knowledge, the term ‘cultural knowledge’ is used. Knowledge about Tyrolean food in Treze Tílias is cultural because it is shared, but also because it distinguishes the Tyrolean migrants from other Brazilians. Furthermore, people in Treze Tílias make use of this knowledge to promote Tyrolean culture and sell specific dishes as cultural attractions to tourists. ‘Cultural knowledge’ does not imply that all members of the group have the same knowledge: ‘Not everything has to be shared for a ‘culture’ to exist. Only enough has to be shared for a people to recognize itself as a cultural community of a certain kind and for members of that community to be able to recognize each other as recipients and custodians of some imagined tradition of meaning and value’ [23].

In the method design and description of results, reference is again made to Barth [22]. According to his framework for analysing knowledge, the following three facets or aspects of traditions of knowledge should be differentiated and discussed: (1) the ‘corpus of substantive assertions and ideas about the world’, (2) the medium that communicates the knowledge—such as words, symbols or actions and (3) the instituted social relations building the framework for the transmission of knowledge [22].

In line with Barth’s suggestions, three aspects of the food knowledge tradition are presented and discussed in this paper: (1) the content of cultural food knowledge, comprising knowledge of the dishes perceived as Tyrolean food. Here, we make use of cultural domain analysis to find out how people interpret the content of the knowledge domain Tyrolean food [24]. (2) The media^3^ through which knowledge is communicated in Treze Tílias, with the focus on dishes in ethnic restaurants in Treze Tílias, and (3) the distribution of knowledge: How are the different media embedded in social relations? And what consequences do these relations have on the transmission of food knowledge and the cultural food knowledge of the group? Changes in food knowledge are then discussed, taking into account historical developments in Treze Tílias.

Although the contents of cultural food knowledge are mentioned, no attempt was made to trace actual changes in particular food habits or the contents of food knowledge from the time of migration to the present day. The focus here was on the transmission of food knowledge and how food knowledge transmission is socially organised in order to understand the mechanisms in the transformation of cultural food knowledge.

## Methods

### Study area and ethnographic setting

Between 1933 and 1938, 789 Austrians, including about 560 Tyroleans, migrated to Treze Tílias, Santa Catarina in southern Brazil [25]. The migration stopped with the outbreak of the Second World War. Between 1938 and the 1960s, the population in Treze Tílias fell due to emigration [26]. In 1963, Treze Tílias became a separate municipality [27] covering an area of 185,205 km^2^. Since the 1980s, the population has grown steadily once again—mainly due to immigration of people from other parts of Santa Catarina. In 2010, the municipality had a population of 6341, of whom 4715 were living in the urban area and 1626 in rural areas. In 2010, there were only 97 people living in Treze Tílias who had not been born in Brazil [28]. Today, the population mainly consists of the descendants of Austrian, Italian and German migrants and Brazilians.

The migration movement goes back to the initiative of the former Austrian Minister of Agriculture, Andreas Thaler. During the global economic crisis, many Austrians were forced to leave their country. Thaler believed that an organised group migration would provide the possibility of establishing a business market and cultural enclave abroad [29]. A location far away from any major city and close to German settlers was chosen to avoid assimilation and preserve culture and language. Thaler formulated selection criteria for the settlers. He chose mainly (Tyrolean) farmers, large families with many children to ensure continuity and craftsmen. All the participants had to be Austrian citizens and of the Roman Catholic faith [30]. Thaler presumed that such a group migration of people sharing the same religious beliefs, ideas and language would contribute to the conservation of their culture [25, 29, 31, 32]. The fact that the original group of migrants stayed together and staunchly maintained the cultural traits of their home country has now formed the basis for the development of ethnic tourism.

Brazil entered the war in 1941 and this had huge consequences for the settlers: the German language was banned, gatherings were prohibited and (cultural) associations were forced to dissolve [33]. While some Tyrolean cultural life always existed in Treze Tílias, the main ‘retrieval’of Tyrolean ‘traditions’ went hand-in-hand with the establishment of ethnic tourism in Treze Tílias. The first hotel in Treze Tílias, *Hotel Austria*, was built in 1948. Back then, the hotel was considered more important for its restaurant as an eating place for workers^4^ and as a meeting point for locals than for its overnight accommodation. A former employee recalls that rice and beans—the Brazilian ‘national dish’ [34], were served every day, together with some kind of meat, such as goulash, schnitzel or roast pork. Apple strudel was also often on the menu. The food was simpler than what is being served in restaurants today, according to a descendant of Tyrolean immigrants. In 1972, *Hotel Tirol* opened [35]. In 1975, only three establishments offering food and/or overnight accommodation existed in Treze Tílias [36].

In the 1970s, the first initiatives were taken to actively promote Treze Tílias as a tourist destination. The anniversary of immigration (*Tirolerfest*), which has been celebrated annually by the migrants since 1934, was turned into a tourist attraction and is now the peak season for tourism each year. In addition to mainly Brazilian tourists, Austrian officials and other Austrian guests—relatives and friends, as well as marching bands and dancing groups—were and continue to be among the visitors. In 1978, the Tyrolean State Governor visited Treze Tílias and promised the migrants ‘cultural assistance and help’. By providing traditional clothes for the local folk groups, he later actively assisted in maintaining Tyrolean culture and establishing ethnic tourism in Treze Tílias, as one Tyrolean immigrant recalls.

In 1980, there were already seven establishments offering food and/or overnight accommodation, with the population of Treze Tílias remaining stable—indicating the rising significance of tourism [37]. In 1981, the tourism secretariat was founded. The first person in charge, a Tyrolean descendant who had lived in Austria for several years, implemented structural changes to transform Treze Tílias into what is now the well-known ‘Brazilian Tyrol’ (*O Tirol Brasileiro*). He facilitated the ‘tyrolification’ of the Treze Tílian cityscape by a new municipal law whereby houses with ‘Tyrolean’ roofs were given tax relief. Owners of existing houses added details such as carved balconies. Official buildings were rebuilt to comply with a Tyrolean cityscape [33]. Slowly, the cityscape changed [38]. Various other projects were implemented, such as the sewing of traditional Tyrolean clothes by women’s groups using a sample brought from Austria. The clothes were initially used to dress the folk groups and later also for hotel staff and staff at the tourist board [38].

In the 1990s, tourism in Treze Tílias received another boost: 41 episodes of the Brazilian *telenovela* ‘*Ana Raio e Zé Trovão*’ [39] were recorded in Treze Tílias, showing the city itself, people in Tyrolean clothes, Tyrolean dancing and music and children singing in German. Ethnic food was also served by Tyroleans to their Brazilian guests—food that is still served as ‘typical’ cuisine in restaurants and hotels.^5^ In 1991, the *telenovela* was broadcast on national television and had the effect of promoting Treze Tílias all over Brazil [33]. Several new hotels were built, such as *Hotel Dreizehnlinden*, *Hotel Alpenrose*, *Hotel Schneider* and *Treze Tílias Parkhotel*. Guesthouses such as the *Pousada Adler*, *Pousada Sítio Palmeiras*, *Pousada das Telhas Azuis* and *Chalé das Termas* also opened. A campsite also operated for a while. The main attractions in Treze Tílias are the cityscape, the food, dancing shows and items by a number of woodcarvers known as *Herrgottschnitzer*.

Since the time of the food shortages faced by early migrants, the food situation in Treze Tílias has changed considerably, not least due to the increased number of tourists. Many restaurants and hotels offer a wide variety of food and new shops have opened, introducing a greater variety of foodstuffs. These structural changes have enabled Tyrolean migrants to enjoy and prepare dishes that are more elaborate than those possible on their arrival in Brazil, and also more elaborate than those they ate in Tyrol before they left Austria, according to interviewees in Treze Tílias. Time did not stand still in Austria either—as mentioned by informants in Tyrol, there has been a change in the food habits in their region since the 1930s towards more elaborate food.

### Study design

A combination of different research methods was used to explore the food knowledge tradition in Treze Tílias (for a summary of the methods used, see Table 1).

**Table 1:** Summary of methods used

Year	Results	Method	Analysis (software)
2008	Historic information about food practice and life in Treze Tílias in 1934	Literature research, qualitative interviews	Qualitative text analysis (Atlas.ti)
2008	Cultural food knowledge in Treze Tílias and Tyrol in the present	Free-Listing	Smith’s Salience index (Anthropac)
2008	Personal network cards showing how food knowledge is transmitted from a personal view	Single name generator and name interpreter	Visualisation (MS Excel, Pajek, SPSS)
2008, 2009	Food practice in restaurants and hotels in Treze Tílias	Participant observation	Qualitative text analysis (Atlas.ti)
2009	Social network: Recommendation network, showing Tyrolean food experts in Treze Tílias	Single name generator	Visualisation of the network (Visone)
2009	Social network: Advice network, indicating actual transmission of knowledge about Tyrolean food in Treze Tílias	Single name generator	Visualisation of the network (Visone)

Letters were analysed to gather contextual information about historical knowledge and developments in the settlement. The letters were written by the first migrants who arrived in Treze Tílias in 1933. Twenty-six of these letters—containing information about the proceedings in the first year of the settlement—were printed in the Tyrolean newspaper ‘*Tiroler Bauernzeitung*’ in 1934. All 26 letters were analysed using Qualitative Text Analysis using Atlas.ti.

***Cultural food knowledge*** was assessed in Tyrol, Austria, and in Treze Tílias, Brazil in 2008 and 2009. We used free-listing [24] to assess whether people perceive “Tyrolean food” (respectively “traditional Tyrolean food”) as a domain, what items are comprised in this domain, and how much people agree on these items. In order to provide the same preconditions for the interviewees, no additional explanation was given to clarify free-listing questions. After asking the free-listing question and after the first listing, redundant questioning was used to maximise free-list output. The free-list items already mentioned were read out and the free-listing question was repeated. The sample size was chosen according to Guest, Bunce & Johnson [40], who state that to explore a shared perception, a sample size of 12 is sufficient to reach data saturation given a narrow research objective and a relatively homogeneous sample.

In *Tyrol, Austria*, 15 interviewees were asked to name ‘all the traditional Tyrolean foods they know’.^6^ Sampling was done using the snowball sampling method. The years of birth of the five male and ten female interviewees were between 1923 and 1973. The interviewees were interviewed in three field sites in Tyrol: the Lower Inn Valley, the Upper Inn Valley and Eastern Tyrol. The Lower Inn Valley (*Wildschönau*) was home to more than 300 of the 789 people who migrated to Treze Tílias. A further 250 migrants originated from other areas of Tyrol, including the Upper Inn Valley and Eastern Tyrol sites [25, 41].

In *Treze Tílias*, *Brazil*, 15 interviewees were asked to name ‘all the Tyrolean foods they know’,^7^ using the same interview technique as in Tyrol, Austria. The eight female and seven male interviewees interviewed came from urban and rural parts of the municipality. All 15 interviewees were Austrian descendants, 13 of them Tyrolean descendants. The two non-Tyroleans lived in close contact with Tyrolean immigrants and their descendants. Their years of birth were between 1921 and 1988. Four interviewees, three women and one man, were born in Austria and migrated to Brazil between 1933 and 1938, while the other interviewees were born in Treze Tílias. The interviews were conducted either in German or in Portuguese, according to the wishes and ability of the individual.

The data obtained in Tyrol and Treze Tílias through this interview technique were analysed using Anthropac [42] to calculate frequency and Smith’s Salience^8^ index of the mentioned items. Items with a higher Smith’s Salience index are assumed to be culturally more important than items with a lower index. The meaning of the particular food terms was explored later during informal talks with informants, participant observation and interviews.

In Treze Tílias, different *media that communicate food knowledge* were accessed and explored in terms of how they are *socially organised.* This was accomplished by (1) visualising personal networks, (2) visualising recommendation and advice networks and (3) exploring food practices in hotels and restaurants in Treze Tílias.

Personal networks [43, 44] were visualised in 2008 with the help of three male and five female informants with a Tyrolean migration background born between 1921 and 1988, using the Wellman approach [45]. A *single name generator*^9^ was used asking respondents to name their sources of food knowledge (referred to throughout this article as ‘alters’): ‘Where does your knowledge come from about the preparation of food, drinks and preserved food?’^10^ The alters may include individuals (e.g. family, friends and acquaintances) and non-individual sources (e.g. television, the internet and cookery books). The alters were specified in more detail by asking *name interpreter* questions to elicit information about age, sex (for individuals only) and relationship ties. The network was visualised on an A0 sheet of paper with the name of the informant in the middle, using pre-defined paper cards which varied in form and colour according to the type of alters (person, textual source, audiovisual source, institution), and in size according to the amount of information received by the informant. The paper cards were assigned by the informant to the alters named, and placed according to emotional closeness to the informant. Frequency of contact was indicated by drawing one, two or three lines or, if there was no longer any contact, by drawing a red line. Once the personal network was completed, the paper cards were glued onto paper and photographed for analysis. The information collected was analysed using MS Excel, Pajek and SPSS 16 [see also 44].

For the recommendation networks about food expertise, 40 people (15 men and 25 women) of different ages were asked in 2009 to recommend somebody who had outstanding knowledge of Tyrolean food and drinks in Treze Tílias.^11^ The same 40 people were asked to name sources from whom they sought advice when they needed information about Tyrolean dishes or drinks.^12^ All 40 interviewees lived in Treze Tílias, but only some of them had a Tyrolean migration background. For visualisation of the network, Visone software was used.^13^

For a closer look at the knowledge transmitted in culinary institutions serving ethnic food from Austria, and how it is transmitted, all the restaurants and hotels in Treze Tílias were visited in 2008 and 2009 to document the dishes offered and how they were promoted. The dishes documented were then compared with the cultural knowledge about Tyrolean food. This was done by visualising the correlation between the number of restaurants offering a special dish and how often that dish was mentioned as a ‘Tyrolean dish’ during the interviews in 2008.

## Results

### The content of the food knowledge tradition: ‘Cultural food knowledge’

In Treze Tílias, the respondents (*n* = 15) listed 234 items representing 144 different dishes when asked to list ‘all the Tyrolean dishes’ they know. The ten culturally most important Tyrolean dishes in Treze Tílias were (1) *Knödel*: a German generic term for dumplings; (2) goulash; (3) *Schmarrn*: a generic term for pancakes, which can be made from flour, milk, eggs, sugar. *Schmarrn* in Treze Tílias is mainly referred to as *Kaiserschmarrn* (see below); (4) *Schnitzel:* a generic term for escalope. In Treze Tílias the term mainly refers to breaded escalope, where the meat can be pork, beef or chicken. Other types of escalope are also known in Treze Tílias; (5) *Spaetzle:* a dish made by boiling small lumps of dough made from flour, water and eggs; (6) *Kaiserschmarrn:* cut-up and sugared thick, fluffy pancake with raisins; (7) *Wienerschnitzel:* breaded veal escalope; (8) *Sauerkraut*: fermented cabbage; (9) *Hackbraten:* meatloaf; and (10) *Schweinsbraten:* roast pork.

In Tyrol, Austria, the respondents (*n* = 15) listed 465 items representing 215 different dishes when asked to list ‘all the Tyrolean dishes’ they know. The ten culturally most important Tyrolean dishes in Tyrol were (1) *Tiroler Knödel*: dumplings made from bread, milk, eggs, flour, spices and some meat and sausage (such as bacon, and/or smoked meat); (2) *Gröstl*: a dish made from potatoes and pieces of boiled meat; (3) *Schweinsbraten*: roast pork; (4) *Muas*: a generic term that can refer to various types of pap; (5) *Schlipfkrapfen*: a special kind of noodles filled with potatoes and cheese; (6) *Leberknödel*: dumplings made from bread, eggs, milk and ground liver; (7) *Kiachl*: a kind of yeast dough fried in oil; (8) *Semmelknödel*: dumplings made from bread, eggs, milk and spices; (9) *Blattln*: dough (of various kinds) fried in oil in a special way; and (10) *Gerstensuppe*: barley soup.

Tyroleans in Austria and Tyrolean migrants and their descendants in Treze Tílias differed in their perceptions of what Tyrolean food comprises. In Tyrol and Treze Tílias together, 306 different Tyrolean dishes were mentioned by the interviewees. Of these dishes, 53 dishes were mentioned at both field sites. The interviewees in Tyrol were in greater agreement on Tyrolean dishes. In Tyrol, seven dishes were named by more than 50% of the interviewees, while in Treze Tílias only two dishes were named by more than 50% of the interviewees.

Comparing the ten most important items according to the Smith’s Salience index of dishes considered Tyrolean in Treze Tílias and Tyrol, only two dishes were mentioned at both field sites: dumplings and roast pork. Dumplings were the most important dish in both areas, mentioned by 11 out of 15 interviewees in Treze Tílias and by 13 out of 15 interviewees in Tyrol. However, the terms and level of abstraction differed between the two groups of interviewees: Tyroleans in Treze Tílias used the generic term dumplings (*Knödel*), while interviewees in Tyrol specified the dumplings as Tyrolean dumplings (*Tiroler Knödel*—dumplings made from bread, milk, eggs, flour, spices and some meat or sausage such as bacon and/or smoked meat). The term *Tiroler Knödel* was used in Treze Tílias by only two interviewees and is therefore not part of cultural knowledge. *Gröstl* (a dish made from potatoes and pieces of boiled meat) was the second most important Tyrolean dish in Tyrol, mentioned by 13 of the 15 people interviewed in Tyrol. In contrast, only one informant in Treze Tílias mentioned this dish.

### Relevant media within the food knowledge tradition

An analysis of personal networks and advice networks revealed that in Treze Tílias, relevant media for transmitting knowledge about Tyrolean food were (a) people (b) textual sources and (c) ethnic restaurants.

#### People

In Treze Tílias, most food knowledge was learned from people directly, mainly from parents, and to a lesser extent from friends and peers. Cooking classes, formal training courses and apprenticeships were also mentioned as sources of information.

Transmitted food knowledge includes information about all kinds of dishes, how to prepare them, and including taste and eating style. In Treze Tílias, women had different functions in food practice then men, requiring different knowledge: everyday, food was usually prepared by women, while the preparation of *churrasco* was a male domain. Women played the greatest role in the transmission of food knowledge to either sex. While women learned almost exclusively from other women, men were more likely than women to learn food knowledge from men. Ninety-two percent of the alters named by female informants in Treze Tílias were women, while women accounted for 63% of alters named by male informants as sources for food knowledge.

**Fig. 1:**
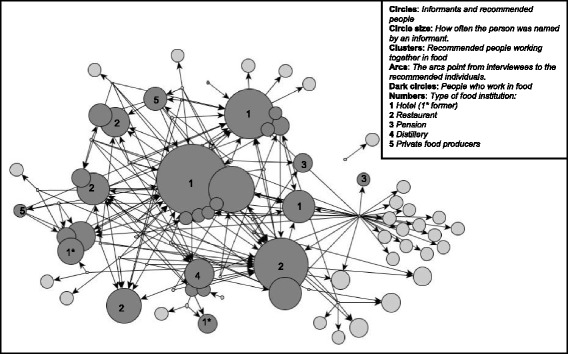
Recommendation network for Tyrolean food knowledge in Treze Tílias (*n* = 40). (Six of the hotels and restaurants identified by the researchers as selling Austrian and Tyrolean food matched the clusters presented in the recommendation network. One hotel that is visible in the network serves food exclusively to its own hotel guests and does not have an open restaurant for Treze Tílians. Another restaurant that serves food to Treze Tílians on special occasions was not mentioned by the informants)

The recommendation networks revealed that Treze Tílians presumed that restaurant or hotel owners and chefs, as well as staff at the local distillery, were the most knowledgeable about Tyrolean food (Fig. 1). In all, the 40 interviewees made 169 recommendations referring to 58 people with outstanding knowledge of Tyrolean food and drink. Seventy-nine percent of the overall recommendations (133 of 169) referred to 29 people who play, or used to play, a key role in selling ‘typical’ food and drinks to tourists. The people mentioned worked in 12 different institutions comprising hotels, restaurants, guesthouses and the distillery. Two of the people recommended were private food producers. The informants provided additional information during the interviews, justifying their list of experts. The reasons stated for choosing people as experts were that the recommended expert was Austrian or of Austrian descendant, ran or worked in a restaurant or hotel serving Tyrolean/Austrian food, frequently travelled to Austria, had professional experience in Austria or completed his or her apprenticeship in Austria and/or came from a family that was considered to value traditions.

The advice networks revealed that informants mostly did not seek direct advice from these experts, despite their knowledge being perceived to be valid information. Informants preferred to ask people who were socially close (family, friends), and almost exclusively asked women when searching for information about Tyrolean food (27 women and only 4 men were indicated). The experts mainly influenced cultural food knowledge via the menus in their restaurants and hotels and at festivities such as the *Tirolerfest*.

#### Textual sources

In Treze Tílias, the textual sources used to gain information about Tyrolean food were very diverse, and the few existing cookery books did not have a wide reach in Treze Tílias: cookery books about Tyrolean food were usually written in German and purchased in Austria due to the lack of purchase options in Treze Tílias.

#### Ethnic restaurants and hotels

An analysis of recommendation networks revealed that ethnic restaurants were places in which cultural food knowledge was transmitted. In restaurants, knowledge was transmitted via the dish itself or via the text in menus and on labels indicating the names of dishes in buffets. Knowledge about taste and smell, colour, ingredients, consistency and arrangement on the plate was transmitted through dishes. Additional information indicating the name and origin of the dish introduced consumers to the concept of ‘typical’, ‘Tyrolean’ and/or ‘Austrian’ food.

In 2009, 19 restaurants were operating in Treze Tílias, 4 of them as part of a hotel. Of those, seven hotels and restaurants were found to serve Tyrolean or Austrian food. In all seven restaurants and hotels, Brazilian and sometimes international food was also offered. The target group was mainly tourists of predominantly Brazilian origin, but the food was not restricted to them. Treze Tílians—regardless of their background (Tyrolean, Italian, German or Brazilian)—also consumed the ‘typical’ food served. During festivities and in restaurants, the food was served to tourists and locals in a similar way.

**Fig. 2:**
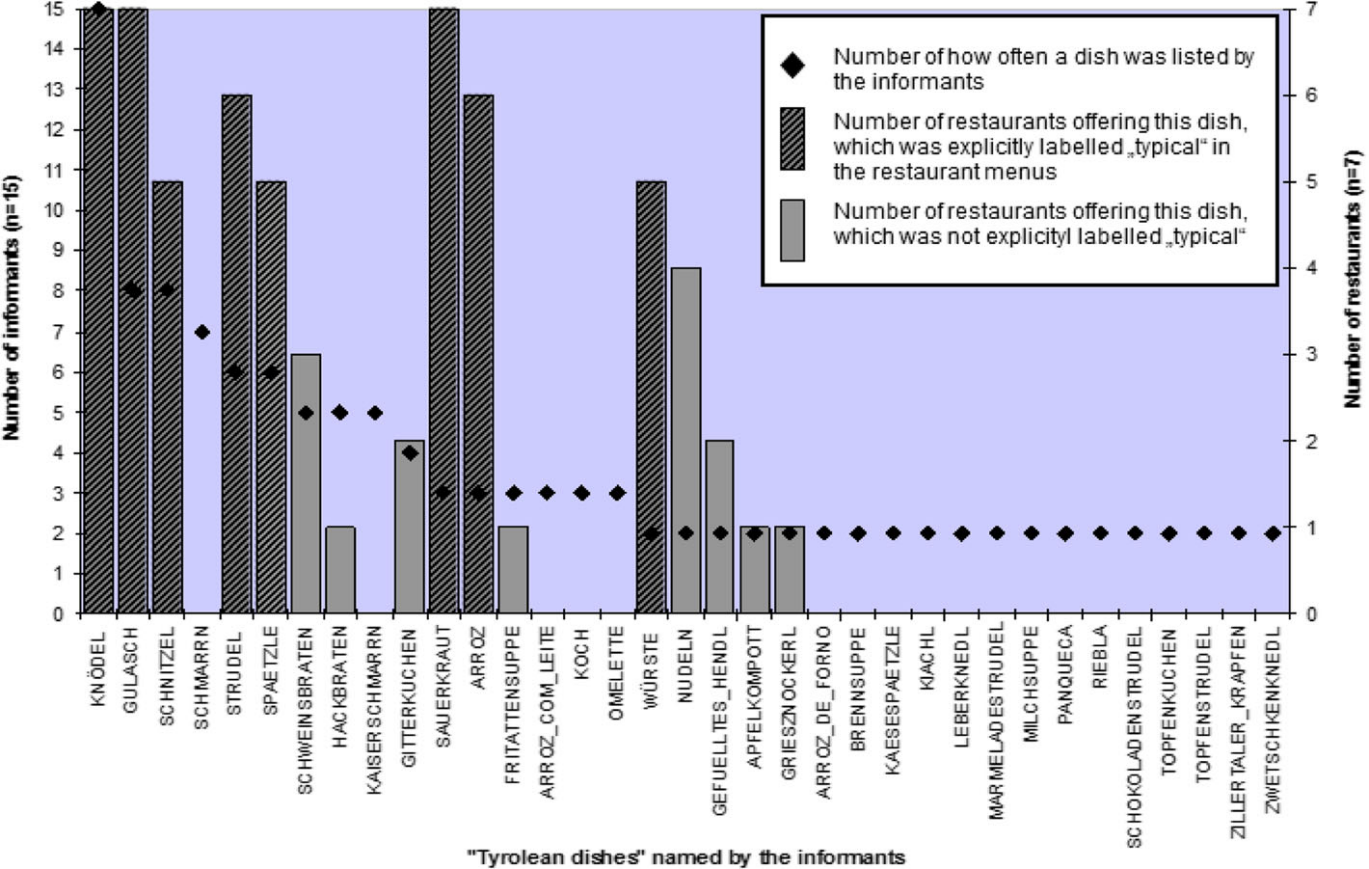
Cultural food knowledge (ranked according to frequency of mention) about Tyrolean food, and frequency of Tyrolean dishes offered in restaurants and hotels. (For the analysis comparing cultural knowledge and food practices, dishes similar in nature were subsumed under generic terms. *Knödelsuppe*, *Semmelknödel*, *Tiroler Knödel* and *Serviettenknödel* were subsumed under the generic term *Knödel* (*Knedl*), *Wienerschnitzel* under the generic term *Schnitzel* and *Apfelstrudel* under *Strudel*. This is why the number of informants mentioning one dish differs in some cases from Table 2.). The figure shows those 35 dishes that were mentioned by 2 or more interviewees as ‘Tyrolean foods’ in Treze Tílias

Dishes served as Tyrolean—or ‘typical’—food in Treze Tílian ethnic restaurants corresponded to the dishes that were commonly perceived as Tyrolean food in Treze Tílias. Overall, of the items mentioned by the Treze Tílian interviewees as Tyrolean dishes, almost 30% were dishes offered in the restaurants and hotels of Treze Tílias. The interviewees were also in greater agreement on those dishes offered in hotels and restaurants than on those not offered. The three dishes mentioned by more than 50% of the interviewees (*Knödel*, *Gulasch* and *Schnitzel*) were also offered in restaurants and hotels—labelled as ‘typical’food (Fig. 2). Comparing the range of products offered in restaurants and hotels with the list provided by interviewees of the culturally most important Tyrolean dishes in Treze Tílias, eight dishes (*Knödel*, *Gulasch*, *Schnitzel*, *Spätzle*, *Wienerschnitzel*,^14^ *Sauerkraut*, *Hackbraten*, *Schweinsbraten*) matched (Table 2, Fig. 2).

**Table 2:** Summary of the culturally most important Tyrolean dishes mentioned by interviewees in Treze Tílias, Brazil (*n* = 15) and Tyrol, Austria (*n* = 15)

Tyrolean dishes in Treze Tílias (*n* = 15)			Tyrolean dishes in Tyrol (*n* = 15)		
Rank by salience	Dish	Smith’s salience	Rank by salience	Dish	Smith’s salience
1	Knödel	0.675	1	Tiroler Knödel	0.699
2	Gulasch	0.37	2	Gröstl	0.687
3	Schmarrn	0.324	3	Schweinsbraten	0.384
4	Schnitzel	0.278	4	Muas	0.373
5	Spätzle	0.272	5	Schlipfkrapfen	0.368
6	Kaiserschmarrn	0.18	6	Leberknödel	0.304
7	Wienerschnitzel	0.164	7	Kiachl	0.291
8	Sauerkraut	0.164	8	Semmelknödel	0.29
9	Hackbraten	0.161	9	Blattln	0.267
10	Schweinsbraten	0.157	10	Gerstensuppe	0.27

Strategies of negotiation with consumers involved (a) framing the culinary experience of the ethnic cuisine with Tyrolean architecture, a specific decor, music and Tyrolean dances and the design of the menu; (b) selecting food that they thought would be well received by Brazilian tourists and other guests, with a few ‘iconic’ dishes, including mostly dumplings, goulash and breaded escalope; and (c) separately identifying these ethnic dishes as a specific category in restaurant menus. The websites^15^ of five Treze Tílian hotels contained the phrases “*pratos típicos austríacos*” (typical Austrian dishes), “*o melhor da cozinha austríaca*” (the best of Austrian cuisine), “*típico tirolês*” (typical Tyrolean), “*comida típica austríaca*” (typical Austrian food) and “*os mais deliciosos pratos típicos da cozinha austríaca*” (the most delicious typical dishes of Austrian cuisine). Dishes were usually indicated in German, and dishes unknown to Brazilian guests were (d) explained separately. Some food was (e) adapted according to the taste of the Brazilian tourists, e.g. the *Schnitzel* served is much smaller in Treze Tílias and would never contain veal, since veal is not acceptable in Brazilian cuisine. Instead, *Schnitzel* was often made from beef, which is not common in Austria. Plates in Treze Tílian restaurants often contained a combination of Brazilian and Austrian food, e.g. rice was often added to ‘typical’ dishes in Treze Tílias, which is also not common in Austria.

Standardised knowledge about some iconic dishes was offered, which could be accessed by everyone interested irrespective of sex, age or cultural background. Through the selection of dishes and eventually transforming dishes to anticipate the taste of Brazilian guests, restaurant staff had transformed the cultural food knowledge of migrants and their descendants in Treze Tílias.

Though ethnic cuisine exists on an institutional level and is cooked in some private homes, Brazilian cuisine is prevalent in Treze Tílias. More restaurants offer Brazilian and international cuisine than Austrian cuisine—reflecting the fact that people with a Tyrolean migration background are a minority population group in Treze Tílias. Austrian cuisine is an important part of Treze Tílian cuisine, but on a quantity basis, it makes up only a fraction of the dishes offered in restaurants and hotels.

## Discussion

### Trajectories of change in the food knowledge tradition of groups of migrants

Cultural food knowledge in Treze Tílias differed from that in Tyrol. The different development in knowledge domains between groups of migrants has been observed before: shared knowledge about medicinal plants differs notably between Tyroleans who migrated to Australia, Brazil and Peru [46]. Also the sources of knowledge and the ways in which knowledge is transmitted differ notably [44]. Vietnamese migrants in Hawaii show increased knowledge about the use of food plants as an immediate effect of adaptation to a new environment [47], and Polish migrants and their descendants enrich and diversify their knowledge about wild edible plants the longer they stay in South America [48]. Descendants of Swiss migrants, 140 years after migration to Colonia Helvetica in Brazil, had abandoned and transformed the dishes of their ancestors, as well as adopted new dishes in their diets [49].

The authors of the above-mentioned studies mainly note external factors to explain the transformation of food knowledge and food habits among migrants. According to them, changes in food habits and food knowledge are the consequence of adaptation to the new environment [47], the prevalence of the predominant cuisine, the tropical climate, the isolation of the migration group from their group of origin, economic factors, extra-ethnic contacts and the accessibility of resources [49] and, in the case of medicinal plants, the predominant healthcare structure and particular sicknesses in the specific area [46]. While the authors of these studies state that external factors impacted food knowledge, they do not explain how these factors actually did so. To close this gap, we first look closely at the impact of the accessibility of resources and the predominant cuisine in Brazil on the knowledge of Tyrolean migrants and their descendants. Then, we relate the outlined social organisation and power relations of food knowledge transmission and distribution of food knowledge in Treze Tílias with the development of tourism.

#### Adapting to the new environment: transforming dishes

Just as the migrants intended to maintain their Austrian traditions in Brazil, they intended to continue cooking their traditional food. Nevertheless, the change of location marked a break in the migrants’ food traditions in terms of how they organised themselves and the food available to them. Immediately after their arrival, the migrants established a large building containing a small store, a mill and a husker for paddy and barley, a storage room for grain and a community kitchen with a dining room [30, 50]. Arriving settlers ate together in this room [30] until the migrant families had built their own houses. Although one settler stated that ‘the same food as in Tyrol’ was cooked in the community kitchen [51], others^16^ provided the information that immediately after their arrival in Brazil, the migrants were forced to radically transform their eating habits in order to survive. Daily life was marked by a shortage of foodstuffs, requiring flexibility and a willingness to cook new foods. Most of the staple foods to which the migrants were accustomed in Austria, such as wheat, rye and milk, were not available, so maize, manioc and sweet potato became the most important staples in their diet. Interviewees stated that the migrants had to learn a lot. Some Brazilian food traditions were welcomed very early on into the Austrians’ kitchens, such as the Brazilian churrasco (mainly beef, roasted on a spit), which the settlers were already preparing themselves in their first years in Brazil. Historical photographs indicate that barbecuing the meat was a male domain [33, 52], which it remains to this day.

When migrants settle in an area where specific ingredients for preparing their traditional meals are absent, they can substitute these ingredients with locally available resources that fulfil the same function, such as taste, flavour or texture within the dish [53]. ‘If specific ingredients of a recipe change, but the presence and organization of required functions are maintained by ingredient substitutions with similar functions, then the structure of the food that is constructed will be similar to the structure of the original food. [...] This would enable a diasporic cultural group to maintain and perpetuate traditional foods’ [54].

Such transformation processes have been shown in Pozuzo, where Tyrolean migrants use cassava instead of potatoes to prepare *Gröstl*, and banana instead of apples to prepare *Strudel* [53]. For example in Treze Tílias, Tyrolean migrants had to substitute rye with maize flour to bake bread in the early years following their arrival. They succeeded only after they had learned from neighbours how to specifically prepare the maize flour for baking bread.

#### Embracing the food habits of cuisines in the country of destination

Tyroleans in Pozuzo, Peru cook dishes from Spanish, Austrian, Creole-Chinese and Andean cuisine [53], Poles in Argentina have been receptive to new food and patterns of consumption influenced by local—mestizo and indigenous—people [48] and Vietnamese immigrants have adapted to the new environment in Hawaii, blending their food knowledge with knowledge from Asian, Pacific and American cuisines [47]. Meanwhile, Tyrolean migrants and their descendants in Treze Tílias have not only integrated Brazilian and international foods into their diet, but favour Brazilian barbecues (*churrasco*) for festivities and embraced culinary habits prevalent in Brazil, changing the rules of how to combine different food elements in their dishes.

Young people embrace the food of the dominant cuisine more easily than the older generation. A change in taste preference from horseradish to chilli among young people in Pozuzo might be leading to the loss of knowledge about horseradish there [53], and in Hawaii, more limited knowledge about Vietnamese food plants was observed among younger Vietnamese migrants [47]. Migrants in Treze Tílias who had been influenced by Tyrolean cuisine in Austria had difficulties in adapting to the range of new and unfamiliar food in Brazil, while their children perceived these new food resources as the norm and continued eating and cooking them.

#### Ethnic tourism and cultural food knowledge

In Treze Tílias, most knowledge about food was transmitted between people. The oral mode of transmission, which is prevalent if knowledge is transmitted between people, fosters a high diversity of food knowledge. ‘Since there is no fixed text from which to correct, variation is constantly creeping in, partly due to forgetting, partly due perhaps to unconscious attempts at improvement, adjustment, creation’ [55].

Textual sources have previously been proven to be a mechanism for ‘correcting’ food knowledge in other research areas, e.g. in India, cookery books helped to constitute a cuisine across different regions by sharing standardised and stereotypical information [56]. But in Treze Tílias, the textual sources used to gain information about Tyrolean food were very diverse. The language barrier excluded a large part of the population, including many descendants of Tyrolean migrants who did not speak German. Due to their diversity and limited accessibility, they were not a factor in generating a shared perception about food. These factors further pointed to a diversity of food knowledge in Treze Tílias, which was reflected in the relatively low agreement about what Tyrolean food comprises in the results about cultural food knowledge.

Ethnic tourism has impacted cultural food knowledge mainly through the establishment of ethnic restaurants, as suggested by the comparison of cultural food knowledge with the food provided in ethnic restaurants. Ethnic restaurants provided Treze Tílians with standardised information about some iconic Tyrolean dishes that were being promoted and thus became widely visible. Anyone interested, irrespective of age, sex and origin, had access to this standardised knowledge about Tyrolean food. The institutionalisation and visibility of Tyrolean dishes combined with accessibility of information created a common understanding of what Tyrolean food comprises.

## Conclusions

Immediately after their arrival in Brazil, Tyrolean migrants had to adapt to a new environment and embraced Brazilian food habits in order to survive. Most food knowledge was transmitted within families, and knowledge about new food resources was learned from German-speaking neighbours. There was no source for transmitting institutionalised and standardised knowledge about Tyrolean food. Even though individual knowledge about Tyrolean food always existed, Tyrolean food acquired a new value with the introduction of ethnic tourism and the commodification of Tyrolean cultural traits. The development of ethnic tourism—and thus the introduction of ethnic restaurants in the culinary landscape—resulted in major changes in the food knowledge tradition of Tyrolean migrants and their descendants in Treze Tílias, Brazil.

In Treze Tílias, many factors influenced the food knowledge of individuals, but ethnic restaurants influenced cultural food knowledge—the common perception of what Tyrolean food is—most. Tyrolean migrants and their descendants had knowledge of different kinds of Tyrolean food, but only a few dishes were commonly perceived as Tyrolean food. This common perception was influenced by the food offered in ethnic restaurants, which was also influenced by tourists’ needs. By providing their customers (tourists and locals) with the concept of ‘Tyrolean’ food, ethnic restaurants have established a structure for classifying food and are therefore a constituting factor for cultural food knowledge. Tyrolean migrants and their descendants in Treze Tílias agreed with each other less on what Tyrolean food comprises than Tyroleans in Tyrol. This was due to the quite recent development of ethnic tourism, the prevailing Brazilian cuisine in Treze Tílias and the relatively limited number of ethnic dishes offered. The distance from Tyrol, the frequent lack of knowledge of the German language and the local setting of being a ‘culinary island’ in Brazil means that Treze Tílians had few resources to check the information they were being given by the ethnic restaurants. This put the staff at ethnic restaurants and hotels in a very powerful position and allowed them to transform the cultural food knowledge of people by introducing new dishes onto their menus. Tourists have had an indirect influence on the cultural food knowledge of the hosts: restaurant staff reacts to tourists’ needs by framing the culinary experience, explaining and eventually transforming dishes.

This study sheds light on knowledge transmission and transformation processes among a group of migrants, explaining why some food traditions are passed down and other traditions are lost. Knowledge traditions develop specifically in different environments and change over time, adapting to new technologies and a changing environment. The food knowledge tradition in Treze Tílias presented here is a snapshot in time. Access to global information via the internet as well as changing social organisation in particular, such as the increased significance of nursery schools, have the potential to transform food knowledge in Treze Tílias and should be considered for further studies. Furthermore, valuable results could be produced by studies focusing on the perspective and knowledge of people without a migration background in Treze Tílias.

## Footnotes

1 According to Godelier [57], identity is the ‘crystallisation within an individual of the social and cultural relations in which he or she is involved and which he or she is led to reproduce or reject’.

2 The term ‘ethnic’ food is mainly used by outsiders to describe a nondominant food practice, for example of a religious or cultural minority group [7, 12].

3 The term ‘media; is used throughout this article for any means for communicating information, including people, visual and textual sources, as well as items such as e.g. dishes in restaurants.

4 Information provided by the tourism office by email on 14 April 2010.

5 In Treze Tílias, Austrian and Tyrolean food prepared in restaurants and hotels is usually labelled as “typical” food by restaurant and hotel owners.

6 The question was asked in German: ‘*Nennen Sie mir bitte traditionelle Tiroler Speisen, die Sie kennen*’.

7 In German, the following question was asked: ‘*Nennen Sie mir bitte alle Tiroler Speisen, die Sie kennen*’. In Portuguese, the following question was asked: ‘*Por favor*, *me diga todos os pratos tiroleses que você conhece’*.

8 ‘Smith’s Salience’ is an index developed by Smith that merges frequency and rank of the mentioned items [58].

9 *Single name generator* means that only one question was asked [45].

10 The question in German was ‘*Woher haben Sie Ihr Wissen zu Speisen, Getränken und Haltbargemachten*?’ and in Portuguese ‘*De onde você adquiriu o seu conhecimento com respeito à preparação de comida, bebida e comida em conservada*?’

11 In German, the following question was asked: ‘*Kannst du mir jemanden empfehlen, der oder die sich besonders gut mit Tiroler Speisen und Getränken auskennt*?’ In Portuguese, the question was ‘*Por favor, pode me recomendar alguém quem sabe muito sobre comidas e bebidas tiroleses*?’

12 In German, the following question was asked: ‘*Woher holst du dir Rat, wenn du etwas zu Tiroler Speisen oder Getränken wissen willst*?’ In Portuguese, the following question was asked: ‘*Quem você consulta se você quer saber alguma coisa sobre comidas e bebidas tiroleses*?’

13 For the visualisation, Visone 2.6.2 was used. Visone software is freeware and can be accessed online at http://visone.info/index.html.

14 The terms *Schnitzel* and *Wienerschnitzel* are often used equivalently by Treze Tílians.

15 This information was gathered in April and May 2011.

16 According to letters from other migrants printed in the newspaper *Tiroler Bauernzeitung* in 1934 and Tyroleans who immigrated between 1933 and 1938.

## References

[1] Kostenzer O, Drewes M (1994). Kleine Kulturgeschichte der Tiroler Küche. Tiroler Küche. Ein Spezialitäten-Kochbuch mit 480 Rezepten und einer kleinen Kulturgeschichte von Otto Kostenzer.

[2] Tolksdorf U, Brednich RW (1994). Nahrungsforschung. Grundriss der Volkskunde. Einführung in die Forschungsfelder der Europäischen Ethnologie.

[3] Tolksdorf U (1975). Essen und Trinken in Ost- und Westpreußen.

[4] Shils E (1983). Tradition (paperback edition ed.).

[5] Hobsbawm E, Hobsbawm E, Ranger TO (1992). Introduction. The invention of tradition.

[6] Linnekin JS (1983). Defining tradition: variations on the Hawaiian identity. Am Ethnol.

[7] Woortmann E, Menasche R (2007). Padrões tradicionais e modernização: comida e trabalho entre camponeses teuto-brasileiros. A agricultura familiar à mesa.

[8] De Garine I (1987). Food, culture and society. The courier.

[9] Yang L, Wall G (2009). Ethnic tourism: a framework and an application. Tourism Manage.

[10] Plano Diretor de Treze Tílias 023/2007. https://leismunicipais.com.br/a/sc/t/treze-tilias/lei-complementar/2007/3/23/lei-complementar-n-23-2007-institui-o-plano-diretor-de-desenvolvimento-municipal-dispoe-sobre-as-normas-fixa-objetivos-e-diretrizes-urbanisticas-do-municipio-de-treze-tilias-e-da-outras-providencias-2017-07-06-versao-compilada. Accessed 28 Mar 2018.

[11] Cohen E, Avieli N (2004). Food in tourism. Attraction and impediment. Annals Tourism Res.

[12] Bak S (2010). Exoticizing the familiar, domesticating the foreign: ethnic food restaurants in Korea. Korea J.

[13] Bertella G (2011). Knowledge in food tourism: the case of Lofoten and Maremma Toscana. Curr Issue Tour.

[14] Sánchez-Cañizares SM, López-Guzmán T (2012). Gastronomy as a tourism resource: profile of the culinary tourist. Curr Issue Tour.

[15] Long LM (2004). Culinary tourism.

[16] Chuang HT (2009). The rise of culinary tourism and its transformation of food cultures: the national cuisine of Taiwan. Copenhagen J Asian Studies.

[17] Bessière J (1998). Local development and heritage: traditional food and cuisine as tourist attractions in rural areas. Sociol Rural.

[18] Everett S, Aitchison C (2008). The role of food tourism in sustaining regional identity: a case study of Cornwall, south West England. J Sustainable Tourism.

[19] Avieli N (2013). What is ‘local food?’ Dynamic culinary heritage in the world heritage site of hoi an, Vietnam. J Heritage Tourism.

[20] Quyen L (2015). The cultural impact of tourism development in a dong Hoa Hiep local community, Cai be district, Vietnam. Asian Soc Sci.

[21] Texeira V, Ribeiro N (2013). The lamprey and the partridge: a multi-sited ethnography of food tourism as an agent of preservation and disfigurement in Central Portugal. J Heritage Tourism.

[22] Barth F (2002). An anthropology of knowledge. Current Anthropol.

[23] Shweder R (2001). Rethinking the object of anthropology and ending up where Kroeber and Kluckhorn began. Am Anthropol.

[24] Bernard R (2002). Research methods in anthropology. Qualitative and quantitative approaches.

[25] Reiter M, Rampl M, Humer A (1993). Dreizehnlinden. Österreicher im Urwald.

[26] Instituto Brasileiro de Geografia e Estatística (1968). Censo Demográfico de 1960. Santa Catarina.

[27] Instituto Brasileiro de Geografia e Estatística. https://cidades.ibge.gov.br/brasil/sc/treze-tilias/historico. Accessed 28 Mar 2018.

[28] Instituto Brasileiro de Geografia e Estatística (2010). Censo Populacional 2010.

[29] Prutsch U. Brasilien - Die Suche nach einer neuen Heimat. Die Auswanderung von ÖsterreicherInnen nach Brasilien 1918-1938. In: Horvath T, Neyer G, editors. Auswanderungen aus Österreich. Von der Mitte des 19. Jahrhunderts bis zur Gegenwart. Wien, Köln, Weimar: Böhlau Verlag; 1996. p. 111–128.

[30] Thaler A. Botschaft aus Amerika. Was die Siedler schon alles geleistet haben. Innsbruck: Tiroler Bauernzeitung; 1934. p. 5.

[31] Hohenbruck O (1954). 50 Jahre Tiroler Bauernpolitik. Geschichte des Tiroler Bauernbundes 1904 bis 1954.

[32] Thaler A (1934). Die österreichische Kolonie Dreizehnlinden in Brasilien.

[33] Reiter M, Osl M, Humer A (2008). 75 Jahre Dreizehnlinden.

[34] Da Matta R (1999). O que faz o brasil, Brasil?.

[35] Hotel Tirol. http://www.webcitation.org/6CSmVoUcH (archived). Accessed 26 Nov 2012.

[36] Instituto Brasileiro de Geografia e Estatística (1981). Censos Econômicos de 1975. Censo dos serviços. Santa Catarina.

[37] Instituto Brasileiro de Geografia e Estatística (1984). IX Recenseamento Geral do Brasil - 1980. Censo dos serviços. Santa Catarina.

[38] Lemos IS (2004). Estratégicas competitivo - cooperativas para o desenvolvimento regional sustentável via turismo - o caso de Treze Tílias – SC. Master Thesis.

[39] Ana Raio e Zé Trovão - Gravações em Treze Tílias. http://www.youtube.com/watch?v=DHa1ZMbxIhQ&feature=related. Accessed 12 Oct 2017.

[40] Guest G, Bunce A, Johnson L (2010). How many interviews are enough?: an experiment with data saturation and variability. Field Methods.

[41] Schabus W, Ernst P, Wiesinger P (1998). Kontaktlinguistische Phänomene in österreichischen Siedlermundarten Südamerikas. Deutsche Sprache in Raum und Zeit: Festschrift für Peter Wiesinger zum 60. Geburtstag.

[42] Borgatti SP, Schensul J, Le Compte M, Nastasi BK, Borgatti SP (1999). Elicitation techniques for cultural domain analysis. Enhanced ethnographic methods. Audiovisual techniques, focused group interviews and elicitation techniques.

[43] Haselmair R, Schönhuth M, Reschke L, Gamper M (2012). Quellen und Weitergabe von Wissen. Eine Untersuchung anhand persönlicher Netzwerkkarten. Zwischen face-to-face und Web 2.0. Mit der Netzwerkperspektive zur Verbindung von Kultur und Struktur.

[44] Haselmair R, Pirker H, Kuhn E, Vogl CR (2014). Personal networks: a tool for gaining insight into the transmission of knowledge about food and medicinal plants among Tyrolean (Austrian) migrants in Australia, Brazil and Peru. J Ethnobiol Ethnomed.

[45] Wellman B (2007). Challenges in collecting personal network data: the nature of personal network analysis. Field Methods.

[46] Pirker H, Haselmair R, Kuhn E, Schunko C, Vogl CR (2012). Transformation of traditional knowledge of medicinal plants: the case of Tyroleans (Austria) who migrated to Australia, Brazil and Peru. J Ethnobiol Ethnomed.

[47] Nguyen ML (2003). Comparison of food plant knowledge between urban Vietnamese living in Vietnam and in Hawai’i. Econ Bot.

[48] Kujawska M, Łuczaj Ł (2015). Wild edible plants used by the Polish community in Misiones, Argentina. Hum Ecol.

[49] Uhle AF, Grivetti LE (1993). Alpine and Brazilian Swiss food patterns after a century of isolation. Ecol Food Nutr.

[50] Thaler A. Botschaft aus Amerika. Es geht gut vorwärts. Innsbruck: Tiroler Bauernzeitung; 1934. p. 5.

[51] Neuhauser G. Botschaft aus Amerika. Brief eines Siedlers. Innsbruck: Tiroler Bauernzeitung; 1934. p. 5.

[52] Benesch L. Dreizehnlinden, die österr. Siedlung in Brasilien 1933–1939. [Private photo album]. Archive of the municipality Wildschönau, Tyrol, Austria; 1939.

[53] Haselmair R. Die Pozuziner Küche. In: Riedmann J, Schober R, editors. Tiroler Heimat. Jahrbuch für Geschichte und Volkskunde Nord-, Ost- und Südtirols. Innsbruck: Universitätsverlag Wagner; 2015. p. 121–7.

[54] Nguyen ML (2007). Community dynamics and functional stability: a recipe for cultural adaptation and continuity. Econ Bot.

[55] Goody J (2000). The power of the written tradition.

[56] Appadurai A (1988). How to make a national cuisine: cookbooks in contemporary India. Comp Stud in Soc History.

[57] Godelier M (2010). Community, society, culture: three keys to understanding today’s conflicted identities. J Royal Anthropol Inst.

[58] Schnegg M, Lang H, editors. Die Analyse kultureller Domänen. Eine praxisorientierte Einführung; 2007. http://www.methoden-der-ethnographie.de/heft3/KulturelleDomaenen.pdf.

